# Monitoring Bridge Dynamic Responses Using Fiber Bragg Grating Tiltmeters

**DOI:** 10.3390/s17102390

**Published:** 2017-10-20

**Authors:** Feng Xiao, Gang S. Chen, J. Leroy Hulsey

**Affiliations:** 1 Department of Civil and Environmental Engineering, University of Alaska Fairbanks, Fairbanks, AK 99775, USA; xfeng2@alaska.edu (F.X.); jlhulsey@alaska.edu (J.L.H.); 2 College of Information Technology and Engineering, Marshall University, Huntington, WV 25755, USA

**Keywords:** fiber Bragg grating (FBG) tiltmeter, vibration monitoring, bridge health monitoring, dynamic identification, expansion bearing

## Abstract

In bridge health monitoring, tiltmeters have been used for measuring rotation and curvature; however, their application in dynamic parameter identification has been lacking. This study installed fiber Bragg grating (FBG) tiltmeters on the bearings of a bridge and monitored the dynamic rotational angle. The dynamic features, including natural frequencies and mode shapes, have been identified successfully. The innovation presented in this paper is the first-time use of FBG tiltmeter readings to identify the natural frequencies of a long-span steel girder bridge. The identified results have been verified using a bridge finite element model. This paper introduces a new method for the dynamic monitoring of a bridge using FBG tiltmeters. Limitations and future research directions are also discussed in the conclusion.

## 1. Introduction

Dynamic feature identification is an important step for the bridge health monitoring. Dynamic data, including natural frequencies, mode shapes, and damping factors, can provide meaningful results if they are used to improve a bridge’s finite element model or conduct damage identification [1,2,3,4]. Traditionally, measurements of the dynamic parameters of structures are based on accelerometers. Sensors, such as the fiber optic accelerometer [5,6], force balanced accelerometer [7,8], and wireless accelerometer [9,10], are typically employed, as they can obtain acceleration directly.

With the development of sensor and measurement technology, other types of sensor, such as strain sensors, GPS, version-based measurement systems, and tiltmeters, can also be used to identify the dynamic features. Fiber optic strain sensors have been used to estimate structural vibration features and have been tested for a beam [11] and model bridge [12] in the laboratory. Moschas and Stiros used GPS to measure dynamic displacements of a bridge, and the modal frequencies were identified [13]. Lee et al. used a version-based dynamic rotational angle measurement system to monitor the dynamic rotational angle of a five-story modal tower [14]. Fukuda et al. developed a vision-based displacement sensor for monitoring the dynamic response [15]. Erol et al. used a tiltmeter to determine the structural dynamic movements of a tall building, and identified the structure’s resonance [16]. This paper includes a bridge vibration study based on the measurement of fiber Bragg grating (FBG) tiltmeters. 

Tiltmeters are a measurement tool for monitoring the health of a bridge. Currently, commercial tiltmeters can provide resolution up to 0.001° [14,17,18]. To monitor the bridge’s overall response, it has been applied for different type of bridges. Zhou et al. installed tiltmeters on the top of a pylon for a cable-stayed bridge to record the rotations of the pylon [19]. Caglayan et al. measured the slope of a bridge deck to identify the vertical displacement of an arch bridge [8]. Helmi et al. measured the rotations and curvature of a box girder bridge to compute the deflection [20]. Norouz et al. installed tiltmeters on the piers of a truss bridge to measure the top of the piers’ displacement [21]. Kim and Laman placed a tiltmeter on bridge abutments and monitored the abutment movements [22]. In conclusion, these measurements were all performed for the purpose of bridge deflection identification. However, monitoring the bridge’s dynamic features based on a tiltmeter’s measurements has been lacking.

In addition to monitoring the bridge’s longitudinal movement in response to a traffic loading and the environment effects [23], this paper studied the dynamic rotational signal from the tiltmeter and identified the bridge’s dynamic features. The innovation is the first-time use of FBG tiltmeter readings to identify the natural frequencies of a long-span steel girder bridge.

## 2. FBG Tiltmeter Technology 

A tiltmeter can not only record the rotation or deflection but can also provide the dynamic features based on signal processing technology [16]. In this study, two FBG-TI-310 (FBG Korea, Inc., Gwangju, Korea) tiltmeters [24] were employed. The fiber optic sensor is a sensing solution using the fiber Bragg grating and measurement system. The theories and characteristics of this fiber optic technology were presented in previous research [25,26]. 

The FBG sensor with very high accuracy can provide non-stop and long-term angle monitoring. The tiltmeter has a measurement range of 8 degrees and has a resolution of ±0.05% FS. A built-in temperature compensator is used to adjust for the effect of temperature on the measurement, besides the rotational angle measurement, the tiltmeter includes a second FBG which provides active temperature compensation [24].

The sampling rate of the FBG tiltmeter is determined by the optical sensing interrogator. The optical sensing interrogator sends wide-spectrum light to optical sensors and indicates the change in the reflected wavelength from the optical sensors. The interrogator selected for this study was the Optical Sensing Interrogator sm130 (Micron Optics, Atlanta, GA, USA), the scan frequency was 1k Hz, and the sampling rate for the FBG tiltmeter was 250 Hz. 

## 3. Bridge Description and Monitoring System

**Figure 1:**
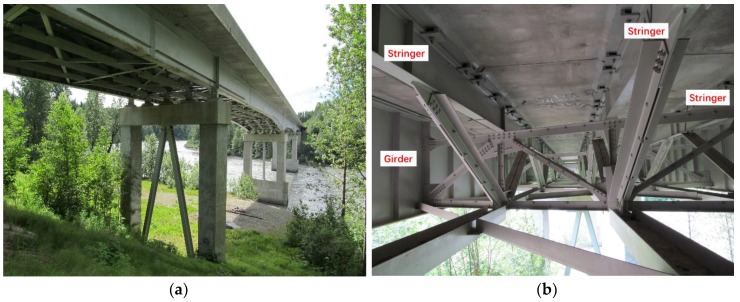

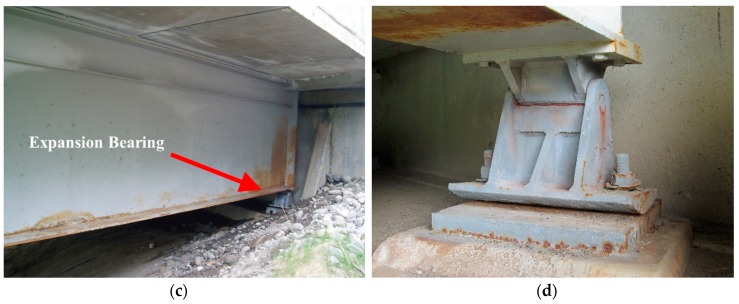
Photographs: (**a**) Chulitna River Bridge; (**b**) Bridge bottom view; (**c**) Expansion bearing at the bridge end; (**d**) Expansion bearing.

The 790-foot Chulitna River Bridge (Figure 1a) is a five-span steel girder plate bridge located in Alaska and is part of the Parks Highway, which links Anchorage with Fairbanks. Heavily loaded vehicles of up to 410,000 pounds regularly travel the route. The bridge deck is 42 feet 2 inches wide with two exterior steel plate girders and three sub-stringers. Three longitudinal steel trusses were installed utilizing the stringers as top chords (Figure 1b). Figure 1c,d shows the bearing condition at the bridge’s end.

**Figure 2:**
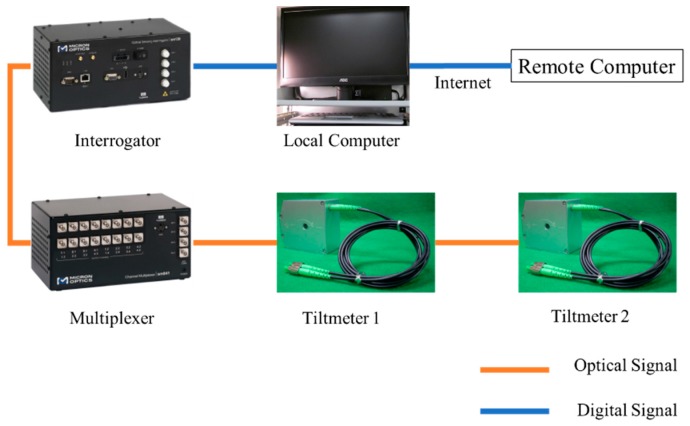
System configuration.

**Figure 3:**
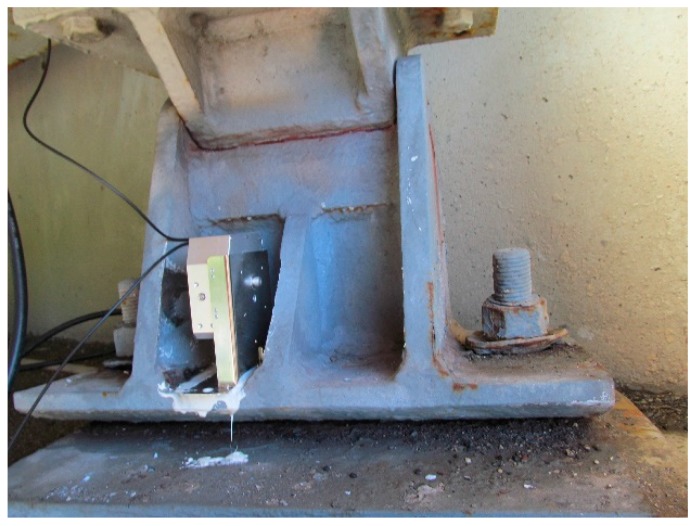
FBG tiltmeter.

The structural health monitoring system consisted of five parts: the sensors, the multiplexer, the interrogator, the local computer, and the remote computer (Figure 2). The interrogator sends optical signals (laser) to the multiplexer, and the multiplexer switches this laser to several lasers, and each laser goes into one optical sensor array. There is an optic Bragg grating in each optical sensor which can reflect a certain wavelength of light back to the interrogator and allow the other wavelengths of light to pass through the sensor, which can be used for the next optical sensor. The interrogator can identify the reflected laser and read the optical signal. The interrogator will store the data in the local computer. The local computer sends the real-time data to the remote computer through the internet. Figure 2 shows tiltmeters 1 and 2 installed in one sensor array. Figure 3 is a photograph of the FBG tiltmeter at the bridge site.

**Figure 4:**
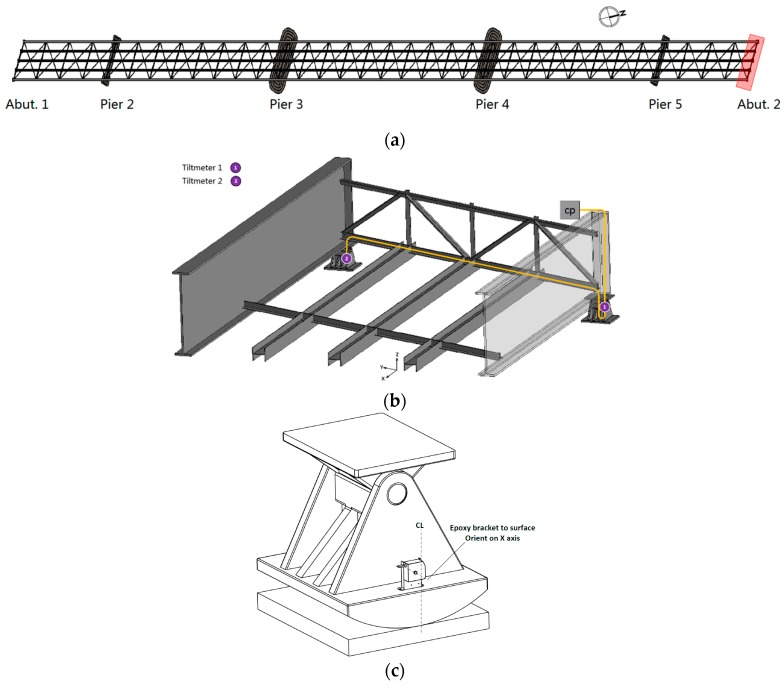
Tiltmeter installation location (**a**) Bridge plan view; (**b**) Abutment 2 view; (**c**) Tiltmeter on expansion bearing.

The tiltmeters were installed on the expansion bearings (Figure 3). Figure 4a shows the expansion bearing location at the abutment 2. Figure 4b shows the details at the abutment 2, and tiltmeter 1 and tiltmeter 2 are connected to the same fiber optical cable and that the cable is connected with the control panel on the bridge site. The control panel communicated with the interrogator and the local computer through the optical signal [23]. 

The expansion bearing is a pin type expansion bearing (Figure 4c), which consisted of a pin at the top that facilitates rotation. The rotation of the bearings can accommodate the bridge’s longitudinal movement. Figure 4c shows the tiltmeter installed on the rocker assembly. The purpose is to monitor the rotation of the rocker assembly in response to the bridge’s longitudinal movement.

## 4. Dynamic Test and Dynamic Properties Identification

**Figure 5:**
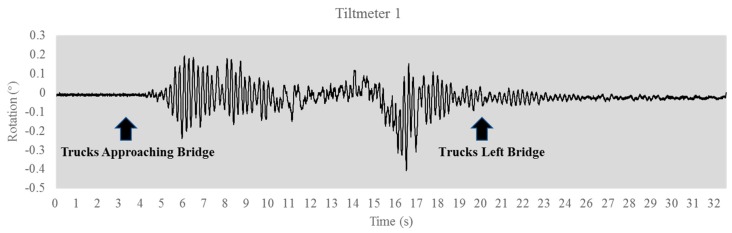
Tiltmeter 1 reading.

**Figure 6:**
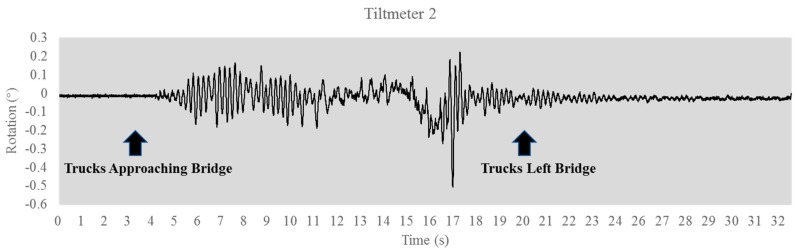
Tiltmeter 2 reading.

A dynamic load test was conducted on the bridge using two dump trucks. The gross weight of those two trucks are weighted before the load test which are 80.3 kips and 82.1 kips. The two trucks were side by side, heading north at a speed of 45 mph. Figure 5 and Figure 6 show the tiltmeter reading in response to the truck loading. The readings can be separated into three phases: trucks approaching the bridge, trucks on the bridge and trucks having left the bridge. In Figure 5 and Figure 6, the trucks arrived at the abutment 2 around at the 17 s, and the bridge is in a free-decay response after the trucks left the bridge, and this study used this free-decay response to identify the bridge’s natural frequencies.

### 4.1. Spectrum Analysis

**Figure 7:**
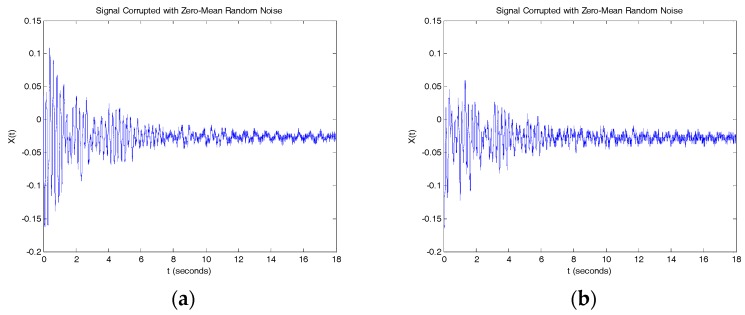
Tiltmeter reading for trucks left bridge. (**a**) Tiltmeter 1 signal; (**b**) Tiltmeter 2 signal.

**Figure 8:**
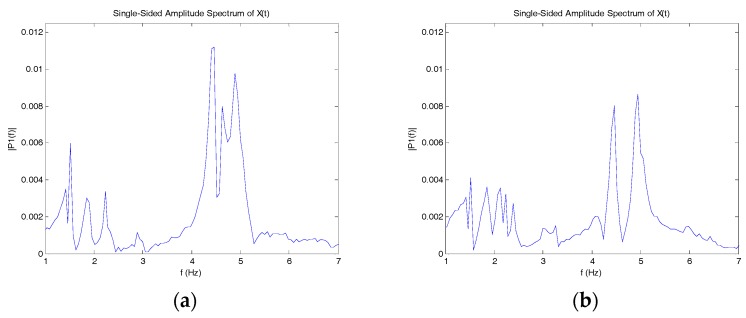
Fast Fourier transform (FFT) of tiltmeter reading. (**a**) Tiltmeter 1 reading; (**b**) Tiltmeter 2 reading.

Figure 7 is the vibration signal after the trucks left the bridge. The dynamic characteristics of the structure were extracted by converting the time domain data to frequency domain data. Figure 8 shows the natural frequencies obtained using the fast Fourier transform (FFT) method. Both sensors yielded 1.50 Hz, 4.47 Hz, and 4.90 Hz as the primary natural frequencies. The identified frequencies can be used as a characteristic quantity or index for damage identification.

**Figure 9:**
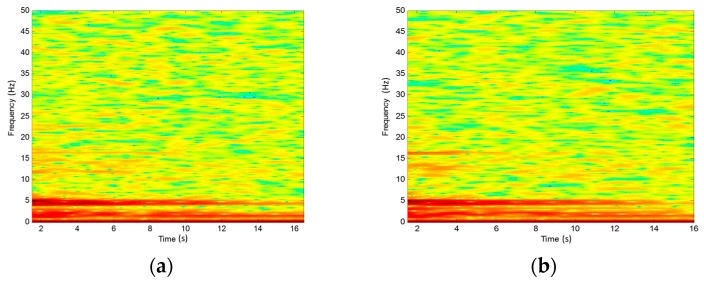
Spectrogram. (**a**) Tiltmeter 1 reading; (**b**) Tiltmeter 2 reading.

To characterize the bridge vibration in the time-domain, time-frequency analysis of the spectrogram is used [27]. Figure 9 shows the spectrogram of the measured signal at the tiltmeter 1 and tiltmeter 2 during the free-decay period; Both Figure 9a,b shows two dominant components. The first component’s corresponding frequencies are 4.47 Hz and 4.90 Hz. The second component’s corresponding frequency is 1.50 Hz. From those results, we can see that all those kinds of spectrum have merged components, the next section will decompose the time-frequency signal.

### 4.2. Modified Empirical Mode Decomposition 

To decompose the signal as being characterized in the time-frequency expression of the signal, we next used the modified empirical mode decomposition method [28] to decompose the tiltmeter 1 reading (Figure 9a). This method was based on empirical mode decomposition (EMD) [29], which established a new framework and avoided the EMD drawbacks [28]. The method decomposed the original signal into a set of elemental signals called “intrinsic mode functions (IMFs)”. 

The decomposed signals are zero-mean components, which must satisfy the following requirements: (1) The number of extreme and the number of zero crossings in the IMF must be equal or differ at most by one; (2) at any point, the mean value of the envelopes defined by the local maxima and local minima must be zero. The signal is locally symmetric around the time axis, and the original signal can now be represented as the sum of *n* IMFs plus a residual $r_{n}\left(t\right)$.

(1) $$x\left(t\right)=\overset{n}{\underset{i=1}{\sum }}c_{i}\left(t\right)+r_{n}\left(t\right)$$

Next, the Hilbert transform is applied to all IMFs, $c_{j}\left(t\right)$, in Equation (1) to derive model parameters.

(2) $$H\left[c_{j}\left(t\right)\right]=\frac{1}{\pi }\int_{-\infty }^{\infty }\frac{c_{j}\left(t\right)}{t-\tau }d\tau$$

The Hilbert transform, $H[c_{j}\left(t\right)]$, and $c_{j}\left(t\right)$ form a complex signal, $Z_{j}\left(t\right)$, where

(3) $$Z_{j}\left(t\right)=c_{j}\left(t\right)+iH[c_{j}\left(t\right)]=a\left(t\right)e^{i\varphi \left(t\right)}$$

Then the envelop of each IMF can be given by

(4) $$a_{j}\left(t\right)=\sqrt{{\left[c_{j}\left(t\right)\right]}^{2}+{\left\{H[c_{j}\left(t\right)]\right\}}^{2}},\;\varphi_{j}\left(t\right)=arctan\left\{\frac{H\left[c_{j}\left(t\right)\right]}{c_{j}\left(t\right)}\right\}$$

in which $a_{j}\left(t\right)$, the instantaneous amplitude of x(t), reflects how the energy of x(t) varies with time. The term $\varphi_{j}\left(t\right)$ is the instantaneous phase of x(t). The instantaneous frequency, ω(t), is defined as the time derivative of the instantaneous phase φ(t) as follows

(5) $$\omega \left(t\right)=\frac{d\varphi \left(t\right)}{dt}$$

Then the original signal x(t) can be expressed as

(6) $$x\left(t\right)=\sum_{j=1}^{n}a_{j}\left(t\right)\exp\left[i\int \omega_{j}\left(t\right)dt\right]$$

The EEMD method is supposed to eliminate the mode mixing problem of EMD, and adds noise to the signal *x*(*t*). The detailed algorithm has been included in Rato et al. [28].

**Figure 10:**
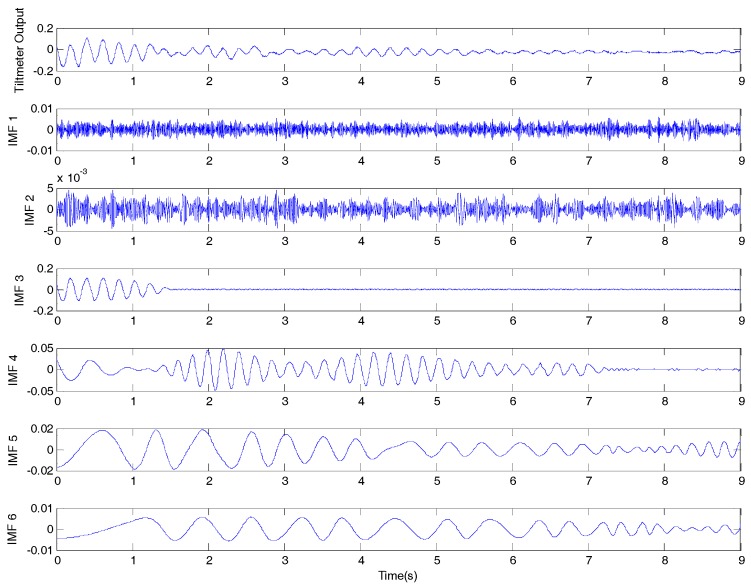
Decomposed components of the tiltmeter 1 reading by using a modified empirical mode decomposition (EMD) method.

Figure 10 shows the decomposed components of the measured tiltmeter 1 reading which included the tiltmeter output and IMF 1 to 6. The damping coefficients can be identified using the slope of the amplitude–time plot [30,31,32].

**Figure 11:**
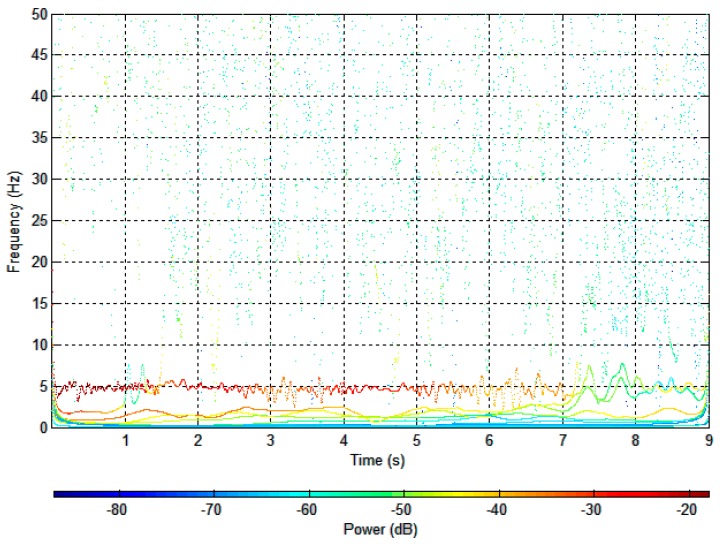
Hilbert spectrum of intrinsic mode functions (IMFs).

To consider the complex frequency properties, the Hilbert spectrum is applied to re-treat the recorded signal. Figure 11 shows the Hilbert spectrum of the decomposed signals in Figure 10. From Figure 11, we can see that those respective components are well separated, and the time-dependent properties of the frequencies are clearly shown. The dominant mode exhibits modulation centered at approximately 4.90 Hz in a red color.

## 5. Discussion and Theoretical Consideration 

To verify the dynamic characteristics obtained from the tiltmeter readings, a bridge finite element model was built using SAP2000 according to the as-built plans. The bridge deck, stringers, and girders are modeled using shell elements; truss members are modeled using frame elements. The bridge has expansion bearings at the abutment 1, pier 2, pier 3, pier 5, and abutment 2; there are fixed bearings at the pier 4. The bridge model’s material properties are based on the construction drawing.

**Figure 12:**
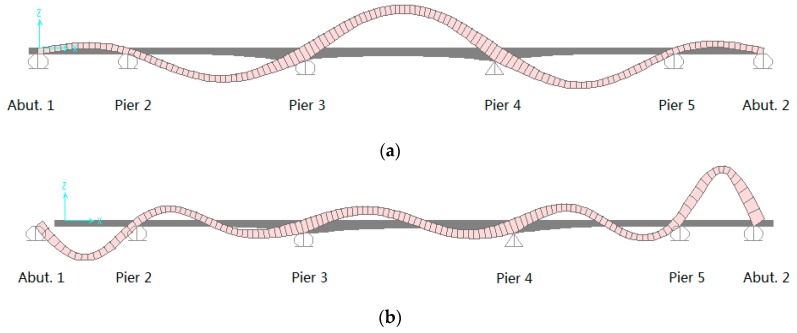

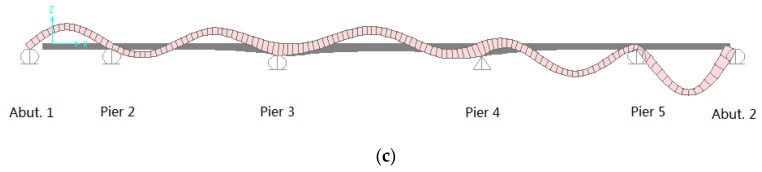
Longitudinal Mode Shapes from Finite Element Method (FEM) Analysis. (**a**) Mode 1 (1.584 Hz; Modal Participating Mass Ratios 0.10); (**b**) Mode 2 (4.348 Hz; Modal Participating Mass Ratios 0.17); (**c**) Mode 3 (5.060 Hz; Modal Participating Mass Ratios 0.18).

Based on the modal participating mass ratios, the first three longitudinal modes have been identified successfully through the numerical mode analysis. Figure 12a is the first mode of longitudinal vibration, Figure 12b is the second mode of longitudinal vibration, and Figure 12c is the third mode of longitudinal vibration. According to the identified mode shapes, there exist large longitudinal displacements at the end bearings for each mode, and they will directly cause the dynamic rotation at the rocker bearings at the abutment 2. The bridge’s finite element model theoretically proved that monitoring the expansion bearings can identify the bridge’s natural frequencies in the longitudinal direction. The longitudinal frequencies can be separated based on the end bearings’ movement. The comparison between the experimental and analytical results are presented in the following section. 

To quantitatively determine the reliability of the natural frequencies obtained from the tiltmeter reading, it was compared with the bridge’s numerical model, and the results are summarized in Table 1. The difference between the field test and the numerical analysis was 5.30% for the 1st mode, 2.81% for the 2nd mode, and 3.16% for the 3rd mode. The use of the FBG tiltmeter makes it possible to acquire the dynamic frequencies with high reliability.

## 6. Conclusions

The tiltmeter reading can extract the primary natural frequencies. This study introduced a new approach to identify long-span steel girder bridge frequencies in the longitudinal direction based on monitoring the dynamic rotational angle at the expansion bearing. The identified frequencies have been compared with the numerical analysis, demonstrating the effectiveness of using a tiltmeter for dynamic feature identification. The analytical and experimental results showed a very strong agreement with a maximum difference of 5.3%. Compared with the accelerometer, the FBG tiltmeter can not only capture the bridge natural frequencies, but also provide the rotational angle.

This study only installed the FBG tiltmeters on the rocker bearings, and it can monitor the swaying movement of the bottom roller. There also exists a rotation of the bearing’s top plate connected to the bridge girder. Future research should install additional tiltmeters on the bridge deck or girder to monitor this rotation which can help to identify the bridge’s vertical dynamic movement, and provide accurate responses of the structure`s bearing. Installing the tiltmeters only on the roller (swaying part of the motion) is not enough, and there should be sensors mounted for measuring the rotation of the deck also. Additionally, the effect of input excitation on the identification results such as road roughness, multiple truck interactions, etc. should also be studied in the next phase of the study.

## Figures and Tables

**Table 1:** Experimental and Analytical Natural Frequencies.

Number of Mode	Analytical Model (Hz)	Test Results (Hz)	Error (%)
1	1.584	1.50	5.30
2	4.348	4.47	2.81
3	5.060	4.90	3.16
